# Trainee resistors: Have our students become our teachers?

**DOI:** 10.1111/medu.15569

**Published:** 2024-10-30

**Authors:** Erin R. Peebles, Rabia Khan

**Affiliations:** ^1^ Division of Pediatric Hospital Medicine, Department of Pediatrics University of British Columbia Vancouver British Columbia Canada; ^2^ Department of Pediatrics, Centre for Health Education Scholarship (CHES) University of British Columbia Vancouver British Columbia Canada

## Abstract

The authors urge medical education to move beyond teaching social determinants, advocating for active resistance to systemic injustices and a more politically engaged and justice‐oriented approach to healthcare training.

The physician as advocate is not a new concept; in fact, many licensing bodies require advocacy as a competency.^1^ Advocacy encourages working within and around the health care system in order to support patients. However, in order to meaningfully change the system, and begin to address social inequities, physicians need to challenge the health system itself. Physicians that focus on addressing social inequities, or fight against oppression, have been conceptualised as engaging in resistance.

Physicians that focus on addressing social inequities, or fight against oppression, have been conceptualised as engaging in resistance.

Physician resistance is defined as ‘…individual and collective expressions of condemnation of social harms and injustices, with the intent of stopping them, preventing them from recurring, and/or holding those responsible for them to account’.^2^ Physicians and trainees who work with patients who are marginalised or oppressed are more likely to engage in resistance.^3^ However, in order to engage in resistance, physicians must stand up to a system that expects obedience and deference.^4^

In this edition, Wyatt et al. paint a compelling picture of acts of trainee resistance over time using the metaphor of a wildfire, whether burning hot, or smouldering under the ground, waiting to re‐ignite. The evolution of the wildfire of resistance is examined through an interplay of contexts, subjectivities and interactions. Trainees who transitioned into positions of power and/or recognition, whether formal or informal, were able to continue active resistance. When the trainee context or subjectivity changed in such a way that the trainee felt unsafe, or somewhat surprisingly, safer, they described engaging in quieter, less explosive acts of resistance. And finally, for one trainee, their resistance effort had succeeded in effecting change and their resistance ‘fizzled’ out.

What is striking in this account of trainee resistors, and other stories of physicians encountering challenging social situations,^5^ is that trainees and physicians seem to feel an individual responsibility to create systemic change. Given the undisputable adverse health outcomes from social injustice, most medical schools now include curricula around the social determinants of health,^6^ and some are beginning to include courses on structural racism.^7^ However, many of these curricula are designed with the assumption that knowing about social determinants will allow physicians to act on social determinants. Trainees enter the workforce with an expectation that they will be able to address the SDH and encounter a system that is focused on efficiency and maintaining the status quo. There is evidence in physician narratives that physicians are educated to feel morally obligated to address the social status of patients, but they are unable to do so.^8^ If left to the individual alone, moral injury or distress results,^9^, ^10^ without change to or for patients.

What is striking in this account of trainee resistors, and other stories of physicians encountering challenging social situations,^5^ is that trainees and physicians seem to feel an individual responsibility to create systemic change.

Current trainees have inherited a health care system based on beliefs and values that may not be aligned with their own, and they are increasingly calling for a dismantling of a system built on epistemic injustice, colonialism and predicated on white supremacy.^11^, ^12^ It is time for a reckoning in medical education. As medical educators, should we continue to offer courses on the social determinants of health that seem to believe just knowing about them is enough to change them^13^? Or do we accept the challenge from the trainee resistors, and change curriculum to support their efforts for change? To what extent are the social determinants of health part of the realities of practicing in a socialised health care system?

To what extent are the social determinants of health part of the realities of practicing in a socialised health care system?

We do not pretend to know the right way to do this, but it is clear that in order to change the system, physicians need to understand it. Instead of offering courses on social determinants of health, often presented as facts, students should be learning about structural racism and oppression that the health system is founded on. There is an entire field of critical pedagogy outlining how to do this.^2^, ^14^ History of medicine courses, which have been pushed to the periphery of curricula, should be revisited with a critical lens, looking at the ways in which the medical system has harmed, and continues to harm, marginalised and oppressed populations. Beyond this, as individual physicians and educators, we have to continue on the path of life‐long learning about these issues and stand in solidarity with our patients and their advocates.

Beyond this, as individual physicians and educators, we have to continue on the path of life‐long learning about these issues and stand in solidarity with our patients and their advocates.

Physicians need to accept that medicine is inherently political and to teach it that way. This requires understanding that physicians in practice do have power and that this power can and should be leveraged for social change. We need to teach that to stay ‘neutral’, to focus only on the biomedical, is to accept the status quo. Trainees can learn to question their own positionality and privilege within these systems through our example and understand that medicine's success has been as a result of the public's trust in us. Physicians should not feel as though the onus for change rests on their shoulders alone but should be taught how to advocate politically, how to speak publicly on divisive topics and how to collaborate with other justice seeking organisations. They should be able to do this without risking professional reprisals from their peers, and those in positions of power within the health system. If we decide as a profession that social equity and justice are within physician purview, as medical educators, we have a duty to teach trainees how to resist effectively and collaboratively, in a way that no trainee feels alone, unsafe or overwhelmed when fighting for social justice. Afterall, the basis of health for all is in the solidarity that comes from standing with others, not in the arbitrary line that separates the doctor from the patient or teacher from student.

Physicians should not feel as though the onus for change rests on their shoulders alone but should be taught how to advocate politically, how to speak publicly on divisive topics and how to collaborate with other justice seeking organisations.

## AUTHOR CONTRIBUTIONS

**Erin R. Peebles:** Conceptualization; writing – original draft. **Rabia Khan:** Supervision; writing – original draft.
